# Unveiling the Novel Benefits of Co-Administering Butyrate and Active Vitamin D3 in Mice Subjected to Chemotherapy-Induced Gut-Derived *Pseudomonas aeruginosa* Sepsis

**DOI:** 10.3390/biomedicines12051026

**Published:** 2024-05-07

**Authors:** Fu-Chen Huang, Shun-Chen Huang

**Affiliations:** 1 Department of Pediatrics, Kaohsiung Chang Gung Memorial Hospital, Chang Gung University College of Medicine, Kaohsiung 833, Taiwan; 2 Department of Anatomic Pathology, Kaohsiung Chang Gung Memorial Hospital, Chang Gung University College of Medicine, Kaohsiung 833, Taiwan; shuang@cgmh.org.tw

**Keywords:** butyrate, vitamin D, chemotherapy, *Pseudomonas aeruginosa*, sepsis

## Abstract

Cancer patients face increased susceptibility to invasive infections, primarily due to ulcerative lesions on mucosal surfaces and immune suppression resulting from chemotherapy. *Pseudomonas aeruginosa* (*P. aeruginosa*) bacteremia is notorious for its rapid progression into fatal sepsis, posing a significant threat to cancer patients, particularly those experiencing chemotherapy-induced neutropenia. This bacterial infection contributes significantly to morbidity and mortality rates among such individuals. Our latest report showed the mutually beneficial effects of postbiotic butyrate on 1,25-dihydroxyvitamin D3 (1,25D3)-controlled innate immunity during *Salmonella* colitis. Hence, we investigated the impact of butyrate and 1,25D3 on chemotherapy-induced gut-derived *P. aeruginosa* sepsis in mice. The chemotherapy-induced gut-derived *P. aeruginosa* sepsis model was established through oral administration of 1 × 10^7^ CFU of the *P. aeruginosa* wild-type strain PAO1 in C57BL/6 mice undergoing chemotherapy. Throughout the infection process, mice were orally administered butyrate and/or 1,25D3. Our observations revealed that the combined action of butyrate and 1,25D3 led to a reduction in the severity of colitis and the invasion of *P. aeruginosa* into the liver and spleen of the mice. This reduction was attributed to an enhancement in the expression of defensive cytokines and antimicrobial peptides within the cecum, coupled with decreased levels of zonulin and claudin-2 proteins in the mucosal lining. These effects were notably more pronounced when compared to treatments administered individually. This study unveils a promising alternative therapy that involves combining postbiotics and 1,25D3 for treating chemotherapy-induced gut-derived *P. aeruginosa* sepsis.

## 1. Introduction

Cancer patients face heightened susceptibility to invasive infections, attributed to ulcerative lesions on mucosal surfaces and chemotherapy-induced immune suppression [1]. Studies conducted in hematology–oncology units have uncovered a significant prevalence of intestinal carriage of *Pseudomonas aeruginosa* (*P. aeruginosa*), ranging from 11.7% to 37% [2]. The extraluminal translocation of intestinal *P. aeruginosa* stands as a crucial pathogenic phenomenon and a significant contributor to systemic infections, particularly in neutropenic patients with hematological malignancies [3]. In immunocompromised hosts, such as individuals experiencing chemotherapy-induced neutropenia, *P. aeruginosa* infections can be linked to a high risk of morbidity and mortality [4].

*P. aeruginosa* bacteremia is notorious for rapidly progressing to fatal sepsis, leading to a high mortality rate, even in previously healthy infants and children [5], or among patients receiving appropriate initial antimicrobial therapy [6]. Given that antibiotic usage poses a risk factor for antimicrobial resistance in *P. aeruginosa,* it is imperative to formulate effective strategies for preventing nosocomial *P. aeruginosa* infections and concurrently minimizing the reliance on antibiotics. Because diet plays a crucial and lasting role in the environmental impact on human health, creating an effective strategy for nutritional management has the potential to address antibiotic overuse and counteract antimicrobial resistance. Moreover, immunotherapy, when administered either alone or in conjunction with antibiotics, holds promise as a viable alternative therapy, offering potential effectiveness in treatment.

Community-acquired *P. aeruginosa* is a reported cause of infectious diarrhea in immunocompromised adults [7] and even immunocompetent children [8,9], sometimes resulting in necrotizing bowel lesions, complicated by fulminant septicemia, and high mortality rates. Remarkably, cytokine responses following intestinal colonization by *P. aeruginosa* were not confined to the intestinal tract but were also observable systemically [10]. Hence, intestinal mucosa, first making contact with the pathogen, have a central part in the innate immune response against *P. aeruginosa* infection. An effective immune response against microbial infection requires the activation of the innate immune response. Yet, the understanding of the innate immune reactions within the intestinal mucosa to *P. aeruginosa* remains largely obscure.

Numerous studies have uncovered a notable occurrence of vitamin D insufficiency among critically ill patients diagnosed with sepsis [11]. For instance, Jeng et al. [12] demonstrated that vitamin D insufficiency was prevalent in 100% of critically ill patients with sepsis, 92% of critically ill patients without sepsis, and 66.5% of healthy controls. A significant association between low serum 25(OH)D concentrations and an increase in blood culture positivity and mortality in the critically ill was found [13]. A systematic review and meta-analysis in adults found an association between vitamin D deficiency and an increased susceptibility to sepsis [14]. Another systematic review and meta-analysis revealed 25(OH)D deficiency in acute and critically ill children is high and associated with increased mortality [15]. Moreover, epidemiological studies have linked vitamin D insufficiency to an increased risk of sepsis [16]. The potential of vitamin D treatment in sepsis syndrome has been explored in animal models, where the administration of 1,25(OH)_2_D3 was associated with improvements in blood coagulation parameters in sepsis [17]. Given the affordability and safety of vitamin D supplementation, further investigation is warranted. Even marginal improvements in sepsis outcomes could have a substantial public health impact.

Probiotics are “Live microorganisms, that when administered in adequate amounts, confer a health benefit on the host” [18]. In our recent findings, we showcased the introduction of probiotics exacerbated gut-derived *P. aeruginosa* sepsis in mice undergoing chemotherapy [19]. Hence, caution has been advised in administering probiotics to patients with acute inflammation. Administration of postbiotics, the soluble factors produced by probiotics, is an evolving therapeutic strategy that would avoid risks associated with the administration of probiotics. Postbiotics are defined as “a preparation of inanimate microorganisms and/or their components that confers a health benefit on the host” [20]. One undeniable advantage of postbiotics is their ability to circumvent the issue of acquiring antibiotic resistance genes and virulence factors, which can potentially occur in vivo with the use of probiotics. The favorable safety profile associated with postbiotics positions them as rational candidates for utilization in functional foods. The UN’s Sustainable Development goals for 2020 included the use of compounds with bioactive properties proposed as a therapeutic strategy due to their stimulating effect on the host’s immune system. Examples of postbiotics include short-chain fatty acids (SCFAs) such as acetate, butyrate, and propionate. They influence various mucosal processes, including absorptive functions, blood flow, mucus release, and cellular differentiation and proliferation. These effects may have clinical significance, particularly in terms of colitis prevention and the restoration of mucosal integrity.

For decades, the gut has been regarded as the motor of sepsis and multiple organ dysfunction syndrome [21]. Any relevant invasive perturbation of the gut likely plays a role in promoting systemic inflammation and infection in the critically ill. The presence of the gut barrier is crucial in preventing excessive immune activation and potentially the development of sepsis [22]. Gut injury can not only propagate local damage and induce intestinal hyperpermeability but can also cause translocation of intact bacteria into the systemic circulation, with subsequent sepsis and organ failure. Permeability, in turn, is controlled by TJ proteins between each cell that regulate the paracellular space [23]. A leaky gut is one of the factors contributing to the development of sepsis [24]. Some individuals have a leaky gut where mucosal integrity is invasion-prone [25], and excessive cytokine production and immune cell adhesion contribute to the development of sepsis. In an inflamed intestine, the ability to decrease inflammatory mediators and enhance epithelial barrier function may be the most critical intervention [26]. Therapies aimed at restoring gut integrity, reversing the pathological effects of barrier dysfunction and optimizing an effective immune response represent exciting avenues of investigation for septic patients in the future.

Preserving barrier integrity is essential for maintaining the gastrointestinal and overall health of the host. Stool butyrate concentrations were significantly lower in the critically ill patients with sepsis in the intensive care unit [27]. Colonization of the intestine with *Roseburia intestinalis*, a bacterium known for producing substantial amounts of butyrate, resulted in a notable decrease in endotoxemia [28], likely attributed to the reinforcement of the intestinal barrier, thus reducing the presence of inflammatory markers like lipopolysaccharide and TNF-α in the serum. The intestinal epithelial barrier, with its intercellular tight junctions (TJs), controls the equilibrium between tolerance and immunity to pathogens. It underscores protective role of postbiotics on sepsis via maintenance of gut barrier integrity.

Hence, in this study, we investigated the impact of postbiotic butyrate combined with 1,25-dihydroxyvitamin D3 (1,25D3) on the innate immune response and barrier integrity of the colon mucosa in mice undergoing chemotherapy-induced gut-derived *P. aeruginosa* sepsis.

## 2. Materials and Methods

### 2.1. Reagents

Butyrate, propionate, and cyclophosphamide were procured from Sigma (St. Louis, MO, USA). 1,25-dihydroxyvitamin D3 (1,25D3), acquired from Biomol Research Laboratories (Plymouth, PA, USA), was stored as a stock solution in pure ethanol at −80 °C in the absence of light. We obtained standard laboratory reagents from Sigma (St. Louis, MO, USA) or Fisher Scientific (Pittsburgh, PA, USA).

### 2.2. Bacterial Strains

The opportunistic pathogen *P. aeruginosa* PAO1-LAC was provided by the Food Industry Research and Development Institute (FIRDI). PAO1-LAC was cultured for 2 h at 37 °C in lysogeny broth supplemented with 50 μg/mL tetracycline. Subsequently, it was diluted at a ratio of 1:100 in fresh broth and sub-cultured for 16 h at 37 °C under gentle aeration. Following this, the bacteria were washed twice and suspended in PBS to achieve a concentration of 10^7^ CFU/mL.

### 2.3. Quantitative Real-Time PCR Analysis of Cecum RNA

Cecal samples were procured and promptly preserved by flash freezing in liquid nitrogen, followed by storage at a temperature of −80 °C. Total RNA was then extracted from both cecal tissue using TRI Reagent (Ambio #15596018, Waltham, MA, USA) and a Directzol RNA MiniPrep kit, following the manufacturer’s instructions. The RNA obtained was converted into complementary DNA (cDNA) through reverse transcription, using a PrimeScript™ RT Reagent Kit (TaKaRa, Cat #RR037A, Kusatsu, Japan). Reverse transcription was conducted in a 20 μL reaction volume, with a final concentration of 1 μg of total RNA. For the subsequent quantitative real-time PCR analysis, the cDNA samples were subjected to the ABI 7500 Real-Time PCR System (Applied Biosystem, Waltham, MA, USA). This analysis utilized the FAST SYBR GREEN MASTER MIX, following the manufacturer’s instructions for optimal protocol execution.

The primers for the mouse genes of interest and reaction protocol were set according to previous reports [29,30,31]. Duplicate reactions were meticulously prepared, and a total of forty amplification cycles were carried out on an ABI 7500 Real-Time PCR System from Applied Biosystems. Each cycle consisted of denaturation at 95 °C for 1 min, annealing at 54 °C for 1 min, and extension at 72 °C for 2 min. The cycle at which the fluorescence surpassed a predefined threshold value during the exponential amplification phase, known as the threshold cycle (Ct), was determined. For subsequent analysis, raw fluorescence data (Rn and DRn) were acquired through the use of the ABI7500 software (SDS V2.3). To standardize the quantity of transcripts, a normalization process was conducted. This process entailed subtracting the mean Ct value of the reference transcript (GAPDH) from the mean Ct value of the target transcript for each experimental condition. The difference between the normalized Ct values of infected and/or treated cells and control cells served as an indicator of alterations in mRNA expression. Throughout the methodology and analysis, careful consideration was given to numerous facets of the MIQE guidelines [28].

### 2.4. Animal Experiments

Gut-derived *P. aeruginosa* sepsis was induced following previously established protocols [32]. The mice used in this study were generously provided by the National Laboratory Animal Center. Specifically, female C57BL/6 mice, aged six to eight weeks and raised in a specific-pathogen–free (SPF) environment at the Kaohsiung Chang Gung Memorial Hospital animal center, were utilized. All animal experiments conducted were in compliance with legal requirements and approved by the Kaohsiung Chang Gung Memorial Hospital Institutional Animal Care and Use Committee. The mice were categorized into five groups: NA (open control), VD (1,25D3 treatment), PS (comparison group), BU (butyrate treatment), and VD + BU (combined treatment with 1,25D3 and butyrate).

Before inducing the sepsis model, mice were orally administered 1,25D3 at a dose of 0.2 μg/25g mice (VD group), butyrate (BU), or a combination of both 1,25D3 and butyrate (VD + BU group) for 3 days via oral gavage. Other groups were fed 100 μL of PBS (open control and PS group). Water and food were provided ad libitum. From the fourth to the seventh day, animals were infected with *P. aeruginosa* PAO1-LAC at a concentration of 10^7^ CFU suspended in 100 μL PBS, or given sterile 1xPBS buffer (100 μL) for the open control group. After infection for 4 days, the mice were again treated with 1,25D3, butyrate, or both for 7 days. Other groups were fed 100 μL of sterile 1xPBS (open control and PS group). On the ninth and twelfth day, mice were given intraperitoneal injections of 150–200 mg/kg of cyclophosphamide (Infection group) or 1xPBS (open control). Following the experimental procedures, mice were humanely euthanized using CO_2_ asphyxiation. Tissue samples from the spleens and livers were collected to assess bacterial colonization. Additionally, samples from the cecum were promptly frozen in liquid nitrogen for mRNA isolation. Some samples were fixed and embedded in paraffin to evaluate disease activity and perform immunohistochemistry (IHC). For survival studies, mice were monitored on weight and clinical score 96 h post bacterial infection (Supplementary Figure S1).

### 2.5. Clinical Scores

The Disease Activity Index (DAI) scores in mice, which provide an overall assessment of their conditions and inflammation, were obtained by evaluating the extent of body weight loss, stool consistency or presence of diarrhea, and the occurrence of fecal occult blood or hematochezia, adhering to a standardized scoring system [33].

### 2.6. Histological Scores

Segments of the ileum, cecum, and colon were prepared for fixation and embedding in paraffin following established protocols. Alternatively, tissue samples can be embedded in O.C.T. compound (Sakura, Torrance, CA, USA), rapidly frozen in liquid nitrogen, and stored at −80 °C. Subsequently, cryosections with thicknesses of 5 or 30 μm were prepared and placed onto glass slides. These sections are then allowed to air dry for 2 h at room temperature before staining with hematoxylin and eosin (H&E). In order to ensure an unbiased evaluation, the tissue sections were anonymized before being assessed by a pathologist. The pathologist assigned scores for the pathological changes, following our previous reports [19].

### 2.7. Immunohistochemical (IHC) Staining

Colon segments were collected and fixed in a solution of 4% paraformaldehyde. Subsequently, 5 µm-thick sections of the colon, embedded in paraffin, were prepared. These sections were then treated to inhibit the activity of endogenous peroxidase by incubating them in a solution of 0.3% hydrogen peroxide in methanol for 20 min at room temperature. For antigen retrieval, the sections underwent treatment with citrate buffer (pH 6.0) and were exposed to three rounds of microwave heating, each lasting 5 min. Following this, the sections were blocked using 5% bovine serum albumin (BSA) at room temperature for 30 min. They were subsequently incubated overnight at 4 °C with primary antibodies including ZO-1 (1:50, Proteintech, Rosemont, IL, USA), Occludin (1:50, Proteintech), and Claudin-1 (1:50, Proteintech). The next step involved incubating the sections with corresponding secondary antibodies at room temperature for 40 min. After washing with phosphate-buffered serum (PBS), the sections were subjected to an avidin–biotin complex, following the instructions provided by the manufacturer of a Boster ABC Kit (Boster, Wuhan, China). To visualize peroxidase activity, diaminobenzidine (DAB) was used as a chromogen. Following this, the sections were counterstained with hematoxylin. Subsequent histological examination was carried out using a Leica TCS SP light microscope (Leica, Heidelberg, Germany).

### 2.8. Statistical Analysis

All of the previously described experiments were conducted precisely, yielding consistent outcomes. Statistical analyses were conducted using appropriate methods: paired Student’s *t*-test was employed for comparing two variables when they were parametric, while the Mann–Whitney U test was utilized for nonparametric comparisons. For comparisons involving three or more nonparametric variables, Kruskal–Wallis one-way analysis of variance was utilized. These statistical analyses were performed using GraphPad Prism 8 software, developed by GraphPad Software in San Diego, CA, USA. A significance threshold of *p* < 0.05 was employed, with results at or below this threshold being deemed statistically significant.

## 3. Results

### 3.1. The Combined Administration of Butyrate and 1,25D3 Effectively Reduces the Severity of Colitis in Mice That Have Undergone Chemotherapy and Subsequently Developed Gut-Derived P. aeruginosa Sepsis

**Figure 1:**
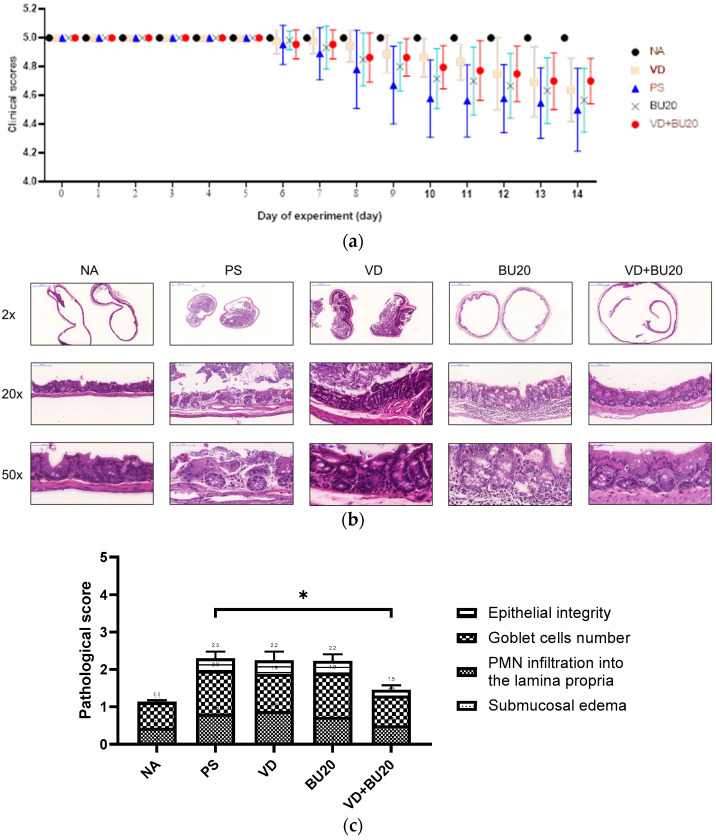
The combined administration of butyrate and 1,25D3 effectively mitigates colitis in mice that have undergone chemotherapy and subsequently developed gut-derived *P. aeruginosa* sepsis. Female C57BL/6 mice aged 6–8 weeks sourced from Charles River, USA, were bred and maintained under specific-pathogen-free conditions at the Center for Cellular and Biomolecular Research in Kaohsiung, Taiwan. These mice were infected with *P. aeruginosa* PAO1-LAC at a concentration of 10^7^ CFU (suspended in 100 μL PBS). An open control group was administered 100 μL of sterile 1xPBS buffer. Prior to and following infection, the mice were orally administered either a vehicle control (5% dimethyl sulfoxide), 1,25D3 at a dosage of 0.2 μg/25g mice/day (VD group), butyrate (BU group), or a combination of 1,25D3 and butyrate (VD + BU group) on a daily basis, as described in the Materials and Methods section. Diarrhea situation scores (**a**) of mice were recorded daily. The cecum specimens were surgically removed, fixed in formaldehyde, and subsequently processed for staining with hematoxylin and eosin (H&E). (**b**) Representative histological images of the cecum from various experimental groups were captured at magnifications of 2×, 20× and 50×. (**c**) Pathological scoring for colitis was conducted based on the assessment of cecum sections obtained from mice in different experimental groups. The data shown are means ± SEM (*n* = 6 mice/group). *, *p* < 0.05.

To investigate the impact of combining 1,25D3 and postbiotics on the severity of colitis in mice afflicted with gut-derived *P. aeruginosa* sepsis, we scrutinized the cecal pathology of infected WT mice in the presence or absence of 1,25D3 (VD), butyrate (BU), or both treatments. As seen in Figure 1a, consistent with a prior study [32], evident pathological alterations were observed in the H&E-stained cecum sections from the infected WT mice (Figure 1b). However, notably contrasting these observations, the combination treatment involving BU and VD significantly mitigated the severity of colitis in mice receiving chemotherapy and affected by gut-derived *P. aeruginosa* sepsis, including diarrhea and pathologic scores. This improvement was evident in various aspects, including reduced body weight loss, improved condition, and decreased pathologic scores in CH57B/6 mice when compared to infection only.

### 3.2. Combination of 1,25D3 and Butyrate Enhanced Anti-Inflammatory Responses and Antimicrobial Peptide Expression in Mice That had Undergone Chemotherapy and Developed Gut-Derived P. aeruginosa Sepsis

**Figure 2:**
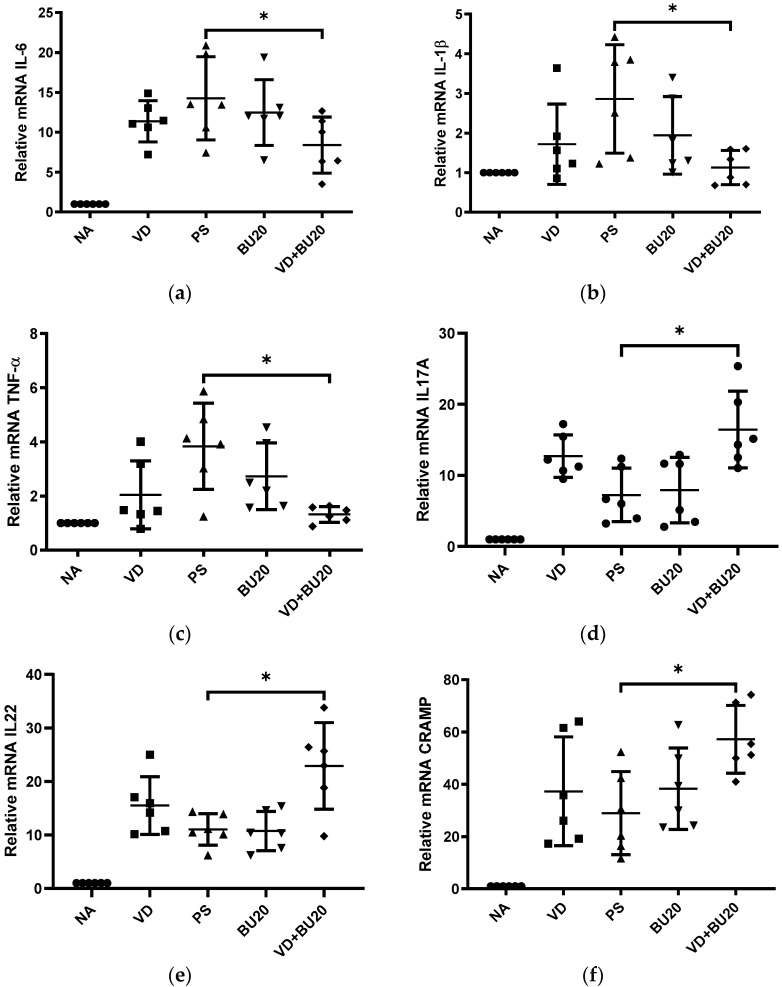

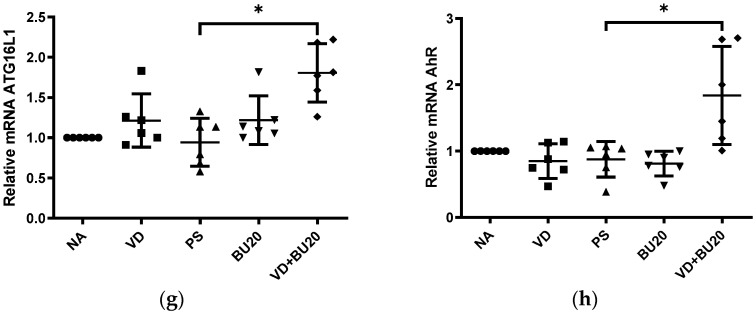
The immunoregulatory effects of combined administration of 1,25D3 and butyrate on cecal cytokines and antimicrobial peptides in mice that had undergone chemotherapy and subsequently developed gut-derived *P. aeruginosa* sepsis. Female C57BL/6 mice, aged 6 to 8 weeks and obtained from Charles River, USA, were bred and housed under specific-pathogen-free conditions at the animal facility within the Center for Cellular and Biomolecular Research in Kaohsiung, Taiwan. Mice were either infected with *P. aeruginosa* PAO1-LAC at a concentration of 10^7^ CFU suspended in 100 μL PBS or given 100 μL of sterile 1xPBS buffer as an open control. Before and after infection, mice received daily oral gavage of vehicle control (5% dimethyl sulfoxide), vitamin D3 at a dose of 0.2 μg/25 g mice/day (VD group), or butyrate (BU group), or a combination of both 1,25D3 and butyrate (VD + BU group), as described in Section 2. Total RNA was isolated from the cecal tissues of the mice. Subsequently, the gene expressions of various markers including IL-6 (**a**), IL-1β (**b**), TNF-α (**c**), IL-17A (**d**), IL-22 (**e**), and CRAMP (a homolog of human cathelicidin LL-37) (**f**), as well as ATG16L1 (**g**) and AhR (**h**) mRNA, were assessed using real-time quantitative PCR. The values obtained were determined as fold increases relative to the levels observed in mice solely infected with *Salmonella* for comparison purposes. The presented data are displayed as means ± the standard error of the mean (SEM) with a sample size of 7 mice per group (*n* = 6 mice/group). An asterisk (*) denotes significant differences observed among the groups, as determined by one-way analysis of variance (ANOVA). *, *p* < 0.05.

To explore the effects of combined 1,25D3 and butyrate on inflammatory and antimicrobial peptide responses in mice subjected to chemotherapy and subsequent gut-derived *P. aeruginosa* sepsis, we assessed the gene expression levels of cytokines and antimicrobial peptides using real-time PCR in cecal tissue obtained from these mice. These assessments were conducted in groups receiving treatment with 1,25D3 (VD), butyrate (BU), or a combination of both (VD + BU). The cecal gene expression of IL-6, IL-1β, TNF-α, IL-17A, IL-22, and CRAMP (homologue of human cathelicidin LL-37) (Figure 2) was significantly elevated in mice with gut-derived *P. aeruginosa* sepsis. In contrast, IL-6, IL-1β, and TNF-α were synergistically suppressed in the cecal tissue of mice that had undergone chemotherapy and developed gut-derived *P. aeruginosa* sepsis but were treated with a combination of active 1,25D3 and butyrate whereas IL-17A, IL-22, CRAMP, ATG16L1 and AhR were synergistically enhanced.

Overall, these findings suggest the synergistic impact of combined 1,25D3 and butyrate treatment on reducing the severity of colitis in mice that have undergone chemotherapy and developed gut-derived *P. aeruginosa* sepsis. This effect is achieved by enhancing antibacterial and anti-inflammatory responses, alongside the potential involvement of ATG16L1 and AhR in mediating these synergistic effects.

### 3.3. Combination of Butyrate and Active 1,25D3 Attenuates Bacterial Translocation in Mice That had Undergone Chemotherapy and Developed Gut-Derived P. aeruginosa Sepsis

**Figure 3:**
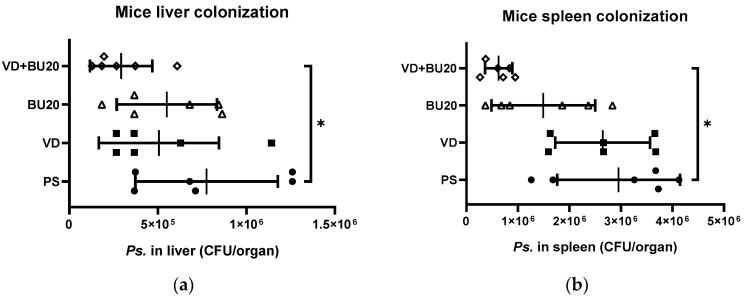
Combination of postbiotic butyrate and active 1,25D3 attenuates systemic translocation of gut-derived *P. aeruginosa* in mice. Female C57BL/6 mice, aged 6 to 8 weeks and obtained from Charles River, USA, were bred and housed under specific-pathogen-free conditions at the animal facility within the Center for Cellular and Biomolecular Research in Kaohsiung, Taiwan. Mice were either infected with *P. aeruginosa* PAO1-LAC at a concentration of 10^7^ CFU suspended in 100 μL PBS or given 100 μL of sterile 1xPBS buffer as an open control. Before and after infection, mice received daily oral gavage of vehicle control (5% dimethyl sulfoxide), vitamin D3 at a dose of 0.2 μg/25 g mice/day (VD group), or butyrate (BU group), or a combination of both 1,25D3 and butyrate (VD + BU group). The quantities of bacteria retrieved from liver (**a**) and spleen (**b**) homogenates of infected and treated mice were measured. The presented data are displayed as means ± SEM (*n* = 6 mice/group). An asterisk (*) denotes significant differences observed among the groups, as determined by one-way analysis of variance (ANOVA). *, *p* < 0.05.

Khailova et al. [34] demonstrated in their study that 1,25D3 has the potential to decrease mortality and systemic bacterial translocation in experimental sepsis in weanling mice. Additionally, they found that 1,25D3 could reduce bacterial translocation in the liver and spleen in a mouse model of gut-derived *P. aeruginosa* sepsis. To further assess the impact of combined treatment involving 1,25D3 and butyrate on tissue bacterial loads, liver and spleen samples were obtained from mice with chemotherapy-induced gut-derived *P. aeruginosa* sepsis that were treated with 1,25D3, butyrate, or a combination of both. These tissue samples were homogenized and plated on LB plates to quantify the colony-forming units (CFU). The results revealed that the combination of 1,25D3 and butyrate led to a reduction in bacterial loads within the liver and spleen of mice that had undergone chemotherapy and developed gut-derived *P. aeruginosa* sepsis (Figure 3).

Altogether, the aforementioned study showed that the combined administration of butyrate and 1,25D3 effectively mitigated the severity of colitis in mice that had undergone chemotherapy and developed gut-derived *P. aeruginosa* sepsis. This effect was attributed to the synergistic impact on anti-inflammatory responses and the inhibition of bacterial invasiveness through enhanced antimicrobial peptide activity.

### 3.4. Combination of Butyrate and Active 1,25D3 Suppressed the Expression of Zonulin and Claudin-2 Protein Expression in Cecal Tissue of Mice That had Undergone Chemotherapy and Developed Gut-Derived P. aeruginosa Sepsis

**Figure 4:**
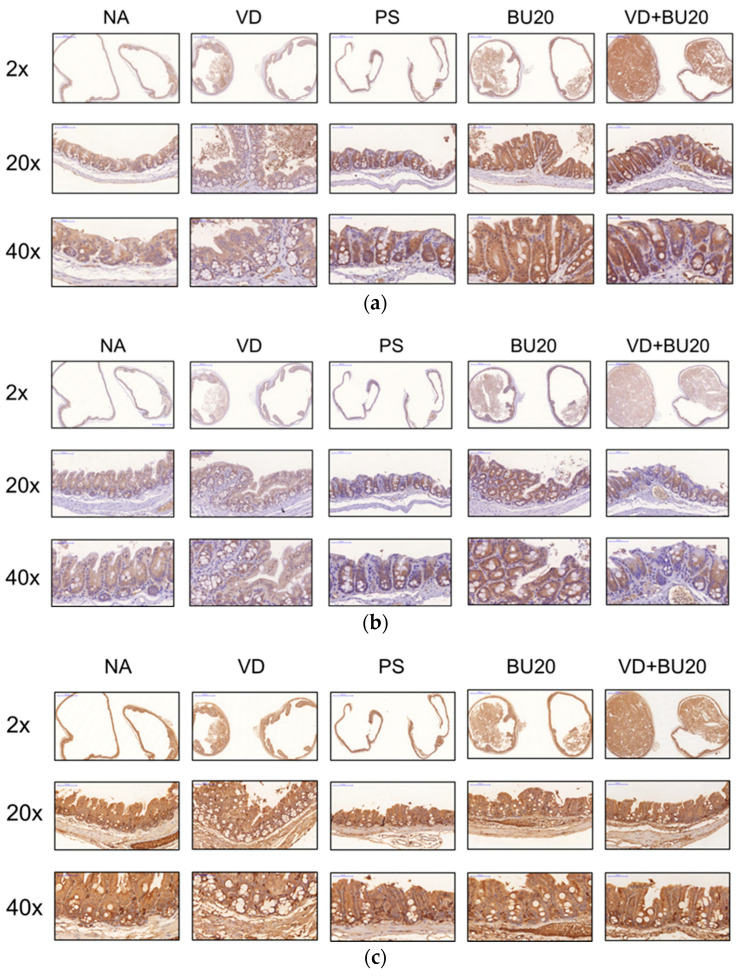

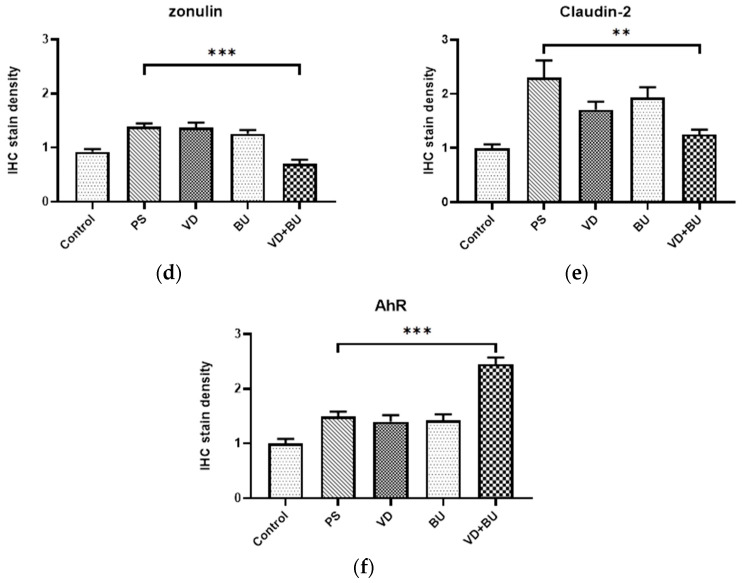
The combined use of butyrate and active 1,25D3 reduced the expression of zonulin and claudin-2 proteins in the cecal tissue of mice that had undergone chemotherapy and subsequently developed gut-derived *P. aeruginosa* sepsis. Female C57BL/6 mice, aged 6 to 8 weeks and obtained from Charles River, USA, were bred and housed under specific-pathogen-free conditions at the animal facility within the Center for Cellular and Biomolecular Research in Kaohsiung, Taiwan. Mice were either infected with *P. aeruginosa* PAO1-LAC at a concentration of 10^7^ CFU suspended in 100 μL PBS or given 100 μL of sterile 1xPBS buffer as an open control. Prior to and after infection, the mice were orally administered either a vehicle control (5% dimethyl sulfoxide), treated with 0.2 μg of 1,25D3 per 25 g of mice per day (VD group), given butyrate (BU group), or received both 1,25D3 and butyrate (VD + BU group) on a daily basis, as described in the Material and Methods section. The detection of zonulin (**a**), claudin-2 (**b**) and AhR (**c**) expression in these groups was performed through immunohistochemistry (IHC) staining (original magnification, 400×; scale bar, 25 µm; *n* = 3). Zonulin (**d**), claudin-2 (**e**) and AhR (**f**) protein expressions in the IHC images were analyzed using ImageJ software (Java 1.8.0_345). ** *p*< 0.01, *** *p*< 0.001.

Altered intestinal permeability, a component of the intestinal barrier, plays a role in many pathological conditions [35], including sepsis. Zonulin-dependent intestinal barrier impairment is an early step leading to altered gut permeability and increased morbidity/mortality in the DSS colitis model [36]. To investigate the role of zonulin and claudin-2 in chemotherapy-induced *P. aeruginosa* sepsis, we performed IHC to detect the expression of zonulin and claudin-2 on the cecal tissue of mice that had undergone chemotherapy and developed gut-derived *P. aeruginosa* sepsis. IHC staining revealed that markedly increased expressions of zonulin and claudin-2 were observed in the cecal tissue of mice with gut-derived *P. aeruginosa* sepsis compared with the untreated mice. However, the combination of butyrate and active 1,25D3 attenuated the increase in zonulin and claudin-2 protein expression in the cecal tissue of mice that had undergone chemotherapy and developed gut-derived *P. aeruginosa* sepsis (Figure 4). These data indicate that a combination of butyrate and 1,25D3 ameliorates bacterial translocation and sepsis in mice that had undergone chemotherapy and developed gut-derived *P. aeruginosa* sepsis by enhancing and maintaining intestinal TJ barrier integrity.

## 4. Discussion

LL-37 is one of the most extensively investigated antimicrobial peptides (AMPs) within the mammalian gene family. Produced by the mucosal epithelium, LL-37 is present in mucosal secretions and plasma, where it demonstrates efficacy in effectively killing a variety of Gram-negative bacteria. It exhibited maximal antibacterial activity [37] against many pathogens. Currently, there is significant interest in AMP inducers, such as 1,25-dihydroxyvitamin D3 and phenylbutyrate, due to their effectiveness in activating the immune system for the treatment of chronic infections [38]. A randomized controlled trial showed substantial benefits of combining oral phenylbutyrate and vitamin D3 therapy in patients with pulmonary tuberculosis [39]. Currently, there is significant interest in AMP inducers, such as 1,25-dihydroxyvitamin D3 and phenylbutyrate, due to their effectiveness in activating the immune system for the treatment of chronic infections [35]. A randomized controlled trial showed substantial benefits of combining oral phenylbutyrate and vitamin D3 therapy in patients with pulmonary tuberculosis [36]. Recent evidence indicates that vitamin D enhances the innate immune response [40,41] by inducing AMPs. Moreover, vitamin D supplementation increases the expression and secretion of antimicrobial peptides against *P. aeruginosa*. Plasma concentrations of LL-37 were positively correlated with those of vitamin D among ICU patients with sepsis [12]. Phenylbutyrate has been demonstrated to induce LL-37-dependent autophagy and facilitate the intracellular elimination of *Mycobacterium tuberculosis* in human macrophages [42].

Chemokines are key mediators of leukocyte recruitment during pathogenic insult that direct the migration of leukocytes throughout the body under both physiological and inflammatory conditions. *P. aeruginosa* is recognized for inducing neutrophilic inflammation in airway epithelial cells, which triggers increased chemokine synthesis, including IL-8, IL-17A [43] or IL-22 [44], consequently leading to neutrophil recruitment to infection sites. This recruitment often results in tissue damage and progressive loss of function. IL-17A and IL-22 enhance basic innate barrier defenses at mucosal surfaces, such as the production of AMPs and neutrophil recruitment; both of these event defend against enteric bacterial pathogens [45]. Colitis-associated epithelial injury and intestinal leakage can be exacerbated in the absence of IL-17A signaling and reveals that IL-17A serves a beneficial role in the intestinal epithelium by helping to maintain the epithelial tight-junction barrier during inflammation [46]. The IL-17 pathway likely plays a critical role in conferring resistance to and modulating the inflammatory response during acute *P. aeruginosa* infection [47,48,49]. Stimulation of macrophages and DCs with IL-17 also contributes to antibacterial immunity, while IL-22 promotes epithelial proliferation and repair following injury [50]. SCFAs can enhance IL-17A expression under T17-inducing conditions [51]. Butyrate supplementation has been shown to increase IL-22 production in intestinal immune cells, thereby mitigating the severity of *Citrobacter rodentium* (*C. rodentium*) infection. This supplementation facilitates the clearance of the pathogen and reduces its spread from the colon to the liver in wild-type (WT) mice [52]. Furthermore, butyrate protects the intestines from inflammation induced by both enteric infection and intestinal injury through the upregulation of IL-22 production [52]. 

1,25(OH)_2_D3 has been shown to diminish the production of pro-inflammatory cytokines/chemokines, including IL-6, TNF-α, IL-1β, and IL-8, subsequent to *P. aeruginosa* infection [53]. Meanwhile, sodium butyrate has shown the ability to alleviate inflammatory responses, reduce neutrophil infiltration and oxidative stress in the lungs, and provide protection against distant acute lung injury resulting from severe burns [54]. This combined action may contribute to establishing a mucosal barrier to prevent the invasion of the intestinal epithelium by pathogenic microorganisms. We observed that the combination of butyrate and 1,25D3 enhanced IL-17A, IL-22, and LL-37 expression but suppressed the expression of proinflammatory cytokines in mice that had undergone chemotherapy and suffered from *P. aeruginosa* sepsis.

Yoseph et al. showed that TJ protein expression is altered in an experimental model of sepsis [55]. Zonulin, the only known physiological modulator of intercellular TJs, is regarded as a master regulator of intercellular TJ in health and disease. The inappropriately increased production of zonulin by enteric pathogens [56] causes a functional loss of barrier function and altered gut permeability, with a subsequent uncontrolled influx of microbial antigens to trigger a submucosal innate immune response [57] and increased morbidity/mortality in the DSS colitis model [36]. This suggests that zonulin acts as a master regulator of intercellular TJ in sepsis. In patients with septicemia, serum zonulin levels were found to be increased [58]. Butyrate has been shown to be a potent regulator of zonulin (decreased serum zonulin concentration in arthritis mice) and the intestinal barrier and appears to be an essential mediator between microbial dysbiosis and barrier function, subsequently attenuating arthritic symptoms [59]. Sodium butyrate has been shown to prevent the lethality associated with severe sepsis and protect against damage to the liver, kidneys, and lungs in a sepsis model induced by cecal ligation and puncture (CLP) [60]. Vitamins D [61] has been observed to modulate the epithelial barrier by reducing serum zonulin levels. Claudin-2 serves as a mediator of the leaky gut barrier during intestinal inflammation [62]. Pore pathway permeability is increased by pore-forming claudin-2 protein upregulation, resulting in barrier loss and the inhibition of wound healing [63]. Claudin-2 is a potential therapeutic target for the modulation of TJ pore and leak pathway permeability. Unfortunately, no drug for claudin-2 modulation currently exists. Butyrate, unlike other short-chain fatty acids (SCFAs), mitigates cytokine-induced barrier dysfunction by reducing the levels of claudin-2 [64], without altering the levels of other tight junction proteins. Intravenous injection of butyrate at a dose of 200 mg/kg ameliorated intestinal injury and improved the survival rate of rats in polymicrobial sepsis by strengthening intestinal barrier function [65] and decreasing bacterial translocation [66]. Dietary sodium butyrate may play an important role in recovering intestinal TJs with a positive effect on maintaining gut integrity [67]. Moreover, LL-37 promotes intestinal barrier integrity accompanied by modulation of the infiltration of neutrophils and monocytes/macrophages in polymicrobial sepsis. Therefore, our observations revealed that the combined treatment of butyrate and 1,25D3 resulted in decreased mucosal expression of zonulin and claudin-2 proteins in the cecum of mice who had undergone chemotherapy and developed *P. aeruginosa* sepsis. This decrease was in contrast to untreated mice, leading to a reduction in the translocation of bacteria to the liver, spleen, and blood.

The findings that AhR spies on bacterial communication, exerts distinct gene expression programs and translates the bacterial signaling vocabulary into the most appropriate host defenses emphasize the crucial role for host AhR as a master regulator of host defense responses, capable of tuning immune defense according to the stage of infection and disease [68]. Butyrate is not a direct ligand for AhR, but it stabilizes AhR, increasing its activity in the presence of true ligands such as microbial pigment virulence factors from *P. aeruginosa*. AhR signaling upregulates IL-22 production to produce antimicrobial peptides [69] and inhibit inflammation and colitis in the gastrointestinal tracts of mice [70]. A recent study also reported that the AhR protects against *P. aeruginosa* infection, as AhR-deficient mice exhibited increased susceptibility to infection [71]. On the other hand, AhR^−/−^ mice are hypersensitive to LPS-induced septic shock, suggesting that AhR may also be involved in immune responses toward other Gram-negative bacteria [72,73,74]. Upon LPS treatment, AhR^−/−^ mice exhibited increased serum levels of IL-6, IL-1β, and TNF-α. In previous studies, we observed the participation of AhR in the collective effects of postbiotics and 1,25D3. This involvement resulted in the suppression of cecal inflammatory responses, downregulation of zonulin and claudin-2 proteins, and an increase in the expression of anti-bacterial IL-22 and LL-37. These combined effects led to a decrease in the severity of colitis and the invasion of *Salmonella* [30,31]. Likewise, we demonstrated that the combination of butyrate and 1,25D3 exerts similar beneficial effects on gut-derived *P. aeruginosa* sepsis in mice receiving chemotherapy, while the inhibition of AhR counteracted these effects. This suggests that AhR is involved in the collective beneficial effects of butyrate and 1,25D3 on cecal inflammation, expression of tight junction (TJ) proteins, and invasion of gut-derived *P. aeruginosa* infection in mice undergoing chemotherapy. In fact, molecular modeling with an AhR ligand binding domain model suggested that vitamin D3 hydroxyderivatives could serve as effective ligands for this receptor [75]. This discovery paves the way for intriguing investigations into the interplay between various vitamin D3 hydroxyderivatives and AhR, as well as the consequent activation of downstream signal transduction pathways. The same is true for melatonin, which can play a beneficial role in the regulation of GI functions [76].

In a *P. aeruginosa* sepsis model, a lack of autophagy protein showed impaired pathogen clearance, decreased survival, and widespread dissemination of bacteria into the blood and lung tissue [77]. Wu et al. reported that *P. aeruginosa* promotes autophagy, suppressing macrophage-mediated bacterial phagocytosis and intracellular killing [78]. Recent research indicates that autophagy positively or negatively regulates the innate immune response in a cell-type-specific manner [79]. Previously, our in vitro study demonstrated that 1,25D3 enhanced VDR-mediated Atg16L1 mRNA and membranous Atg16L1 protein expression, leading to enhanced autophagic clearance of *Salmonella* in intestinal epithelial cells but suppressed IL-1β expression [80]. Likewise, this study observed that a combination of 1,25D3 and butyrate enhanced the autophagy gene ATG16L1 but suppressed proinflammatory IL-1β mRNA expression in chemotherapy-receiving mice complicated with gut-derived *P. aeruginosa* sepsis.

### Limitations

While the therapeutic potential of active vitamin D is promising, its clinical use has been limited by the risk of hypercalcemia, a condition characterized by elevated levels of calcium in the blood. However, a systematic review of existing studies [81] did not find evidence to suggest that vitamin D or its analogues worsen colitis or pose harm to participants. This indicates that they are generally well tolerated in the context of colitis treatment. To address concerns regarding hypercalcemia, there is growing interest in developing vitamin D analogues that exhibit selective binding to the vitamin D receptor (VDR) without inducing elevated calcium levels. These selective VDR ligands could offer the therapeutic benefits of vitamin D while minimizing the risk of adverse effects associated with hypercalcemia. Such advancements hold promise for improving the safety and efficacy of vitamin-D-based therapies for various conditions, including colitis. Indeed, while animal studies provide valuable insights into the potential effects of interventions, their translation to human applications requires cautious interpretation. Experimental models serve as a critical platform for exploring the relationships between various interventions and disease processes, providing a basis for further investigation. Moving forward, there is a clear need for more clinical trials involving human participants to elucidate the impact of combining postbiotics and vitamin D on infection outcomes. These trials can help determine the optimal dosages and formulations of both postbiotics and vitamin D necessary to achieve synergistic effects in humans. By conducting rigorous clinical research, we can better understand how these interventions may benefit human health and inform future therapeutic strategies.

## 5. Conclusions

*P. aeruginosa* has the highest fatality rate among Gram-negative bacteria and is also the most common infection in the hospital. The generation of multi-drug resistant strains also exacerbates this situation. This study explores, using a gut-derived *P. aeruginosa* sepsis animal model, the benefits of using postbiotics plus VD in *P. aeruginosa* sepsis in patients receiving chemotherapy and the mechanisms of gut-derived *P. aeruginosa* sepsis, particularly focusing on the interaction of AhR, innate immunity and autophagy in colon mucosa. This strategy could be used to reduce the abuse of antibiotics and waste of resources in hospitals, increase the productivity of family society, reduce economic depletion, and significantly contribute to the global fight against multi-drug resistant *P. aeruginosa* infection. We can extend this treatment to the study of various infections. Since AhR has been found to control a variety of genes, we can further investigate its epigenetic regulation and disease correlation to achieve preventive medicine and personalized treatment goals.

## Data Availability

The data presented in this study are available on request from the corresponding author.

## Supplementary Materials

The following supporting information can be downloaded at: https://www.mdpi.com/article/10.3390/biomedicines12051026/s1, Figure S1: Experimental groups and protocol.
