# Supplementary material for: Unveiling the Novel Benefits of Co-Administering Butyrate and Active Vitamin D3 in Mice Subjected to Chemotherapy-Induced Gut-Derived Pseudomonas aeruginosa Sepsis
Source: Biomedicines. 2024 May 7;12(5):1026. doi: 10.3390/biomedicines12051026 (PMC11118095; doi:10.3390/biomedicines12051026)
Supplement: Supplementary file 1 [file biomedicines-12-01026-s001.zip › biomedicines-2954422-supplementary.pdf]

Figure S1. Experimental protocol

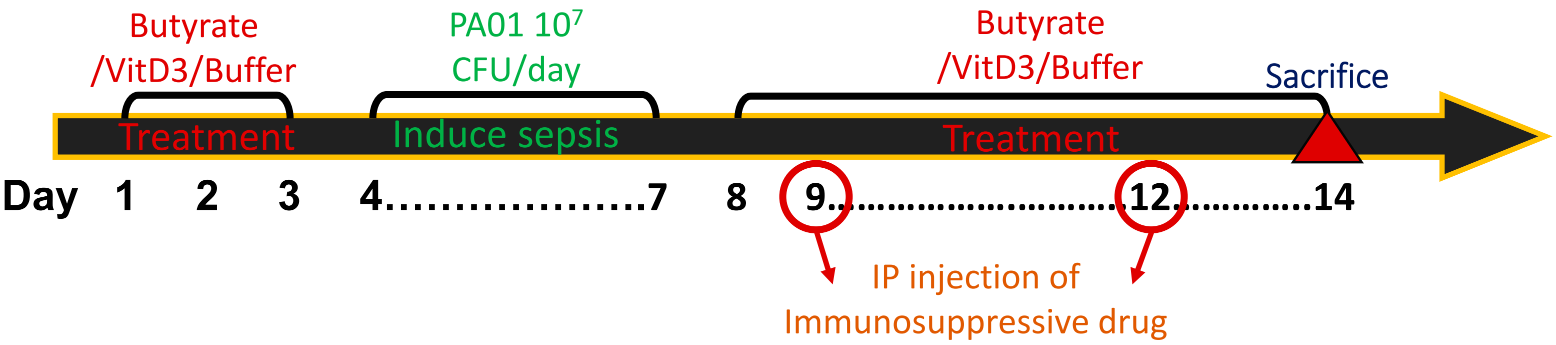

Postbiotics: 20 mg sodium butyrate/kg mice per day
VitD3: 0.2 μg /25 g mice per day
Buffer: 1xPBS (used as vehicle)
PA01: 10^7 CFU *Pseudomonas aeruginosa* PAO1-LAC
Immunosuppressive drug: 4 mg/20 g mice cyclophosphamide
